# Cerebrospinal fluid antibodies to aquaporin-4 in neuromyelitis optica and related disorders: frequency, origin, and diagnostic relevance

**DOI:** 10.1186/1742-2094-7-52

**Published:** 2010-09-08

**Authors:** Sven Jarius, Diego Franciotta, Friedemann Paul, Klemens Ruprecht, Roberto Bergamaschi, Paulus S Rommer, Reinhard Reuss, Christian Probst, Wolfgang Kristoferitsch, Klaus Peter Wandinger, Brigitte Wildemann

**Affiliations:** 1 Division of Molecular Neuroimmunology, Department of Neurology, University of Heidelberg, Heidelberg, Germany; 2 IRCCS, Foundation "Neurological Institute C. Mondino", University of Pavia, Pavia, Italy; 3 NeuroCure Clinical Research Center, Charité - University Medicine Berlin, Berlin, Germany; 4 Department of Neurology, Charité - University Medicine Berlin, Berlin, Germany; 5 Department of Neurology, Medical University of Vienna, Vienna, Austria; 6 Department of Neurology, Hospital Hohe Warte, Bayreuth, Germany; 7 Institute for Experimental Immunology, affiliated to Euroimmun, Luebeck, Germany; 8 Department of Neurology, Sozialmedizinisches Zentrum Ost - Donauspital, Vienna, Austria; 9 Institute for Neuroimmunology and Clinical MS Research, University Medical Center Eppendorf, Hamburg, Germany

## Abstract

**Background:**

In 70-80% of cases, neuromyelitis optica (NMO) is associated with highly specific serum auto-antibodies to aquaporin-4 (termed AQP4-Ab or NMO-IgG). Recent evidence strongly suggests that AQP4-Ab are directly involved in the immunopathogenesis of NMO.

**Objective:**

To assess the frequency, syndrome specificity, diagnostic relevance, and origin of cerebrospinal fluid (CSF) AQP4-Ab in patients with NMO spectrum disorders (NMOSD).

**Methods:**

87 CSF samples from 37 patients with NMOSD and 42 controls with other neurological diseases were tested for AQP4-Ab in a cell based assay using recombinant human AQP4. Twenty-three paired CSF and serum samples from AQP4-Ab seropositive NMOSD patients were further analysed for intrathecal IgG synthesis to AQP4.

**Results:**

AQP4-Ab were detectable in 68% of CSF samples from AQP4-Ab seropositive patients with NMOSD, but in none of the CSF samples from AQP4-Ab seronegative patients with NMOSD and in none of the control samples. Acute disease relapse within 30 days prior to lumbar puncture, AQP4-Ab serum titres >1:250, and blood-CSF barrier dysfunction, but not treatment status, predicted CSF AQP4-Ab positivity. A positive AQP4-specific antibody index was present in 1/23 samples analysed.

**Conclusions:**

AQP4-Ab are detectable in the CSF of most patients with NMOSD, mainly during relapse, and are highly specific for this condition. In the cohort analysed in this study, testing for CSF AQP4-Ab did not improve the sensitivity and specificity of the current diagnostic criteria for NMO. The substantial lack of intrathecal AQP4-Ab synthesis in patients with NMOSD may reflect the unique localisation of the target antigen at the blood brain barrier, and is important for our understanding of the immunopathogenesis of the disease.

## Background

Neuromyelitis optica (NMO) is a rare inflammatory disorder of the central nervous system (CNS) that predominantly affects the optic nerves and the spinal cord [1]. Recent evidence suggests that serum antibodies to aquaporin-4 (AQP4-Ab or NMO-IgG) are directly involved in the pathogenesis of NMO [2-8]. Only very little is known to date about frequency, titres, diagnostic relevance, and pathogenic impact of AQP4-Ab in the cerebrospinal fluid (CSF) of patients with NMO and related disorders. Most previous studies investigating AQP4-Ab did not evaluate CSF,[2,5,9-11] used assays with low sensitivity,[12] or did not take into account blood-CSF barrier dysfunction [13]. In particular, it is still widely unknown whether AQP4-Ab is produced intrathecally. It is also unclear whether testing of CSF for AQP4-Ab helps to diagnose NMO in patients negative for serum AQP4-Ab as suggested by a recent case report [12]. To address these issues, we tested a large series of consecutive, paired CSF and serum samples from Caucasian patients with NMO or its *formes frustes*, longitudinally extensive transverse myelitis (LETM) or optic neuritis (ON), as well as from controls for AQP4-Ab using a recombinant cell based assay [14]. In contrast to a previous study,[13] we controlled for possible disturbances of the blood-CSF barrier.

## Patients and methods

87 CSF samples were analysed, including 45 samples from 37 patients with NMO spectrum disorders (NMOSD) from Germany, Austria, and Italy (serum AQP4-Ab positive in 31, negative in 14 samples) and 42 samples from control patients with multiple sclerosis (MS) or other neurological diseases (OND) (Table 1). NMOSD samples included 8 follow-up samples (AQP4-Ab seropositive in 7, seronegative in 1) from 8 different patients. 29/40 (73%) CSF samples in the NMOSD group were obtained within 30 days from onset of the most recent clinical attack (myelitis in 17; ON in 8; myelitis and ON in 4); in 5 cases the date of relapse onset was not exactly known. Median time between onset of relapse and LP was 14 days (range, 2-640), and 8.5 (2-30) if only samples taken within 30 days after relapse onset were considered. 31/38 samples from control patients with inflammatory disorders (81.6%) were taken within 30 days after onset of attack; 5 were in remission at time of LP; and 2 had chronic progressive disease. Median disease duration was 19.4 months (range, 0.1-286) in the NMOSD group and 19.3 (0.1-143.5) in the MS group. Lumbar puncture was done for diagnostic purposes in all cases. Samples were stored at -80°C until tested. All samples were tested in a cell based assay (CBA) as previously described [14]. This assay was previously demonstrated to have a sensitivity of 78% for NMO and a specificity of 100% [14]. From 23 paired CSF and serum samples sufficient material was available for assessment of the AQP4-specific antibody index (AI), AI_AQP4_. Calculation of AIs allows quantification of antigen-specific intrathecal antibody synthesis [15,16]. Briefly, AI_AQP4 _values were calculated as the ratio between the CSF/serum quotient for AQP4-IgG, Q_AQP4_, and the CSF/serum quotient for total IgG, Q_IgG_; i.e., AI_AQP4 _= Q_AQP4_/Q_IgG_. If AQP4-IgG are produced intrathecally, Q_AQP4 _would exceed Q_IgG _, resulting in AI values >1. Usually, values >1.5 are considered as evidence of intrathecal specific antibody synthesis [15,16]. However, if titres instead of concentrations are used to calculate the AI, a cut-off of 4 has been recommended [17]. Reiber's empiric hyperbolic function Q_lim _was applied to control for possible underestimation of intrathecal specific synthesis due to disturbances of the blood-CSF barrier function:[18]

$${\text{Q}}_{\text{lim(IgG)}}=0.93\sqrt{{\left({\text{Q}}_{\text{AIb}}\right)}^{2}+6\times 10^{-6}}-1.7\times 10^{-3}$$

In case of Q_IgG _> Q_lim(IgG)_, AI_AQP4 _was calculated as the ratio between Q_AQP4 _and Q_lim(IgG)_, i.e., AI_AQP4 _= Q_AQP4_/Q_lim(IgG)_. For assessment of the AI, serum samples were tested at 1:10, 1:100, 1:500, 1:1000, and 1:5000 dilutions, and at 1:25, 1:50, 1:62.5, 1:75, 1:125, 1:250, 1:750, 1:1250, 1:1750, 1:2000, 1:2500, 1:3000, 1:4000, 1:6000, 1:7000, 1:8000, 1:9000, and 1:10000 dilutions, where applicable. CSF samples were tested undiluted, at 1:10 dilution, and, in addition, at dilutions that would indicate intrathecal production as defined by an elevated AQP4-AI of >4.

Values for Q_IgG _exceeding the hyperbolic discrimination line, Q_lim_, or detection of CSF-restricted oligoclonal bands (OCB) (data taken from the patient records), were considered as indicative of intrathecal synthesis of total IgG (as opposed to AQP4-specific IgG) [16]. The CSF/serum albumin ratio, Q_Alb _= Alb_CSF_[mg/l]/Alb_serum_[g/l], was used to assess the blood-CSF barrier function. The upper reference limit of Q_Alb _was calculated as 4+(*a*/15) with *a *representing the patient's age [19].

**Table 1:** Epidemiological data and sample numbers.

	Number of patients	Caucasian	Sex ratio, male: female	Relapsing course	Paired CSF/serum samples	Median age at LP (range)	Untreated at time of LP (%)
Total	79	72/79 (91)	1:4.6	59/79 (75)	87	40 (15-72)	56/80 (70)
NMOSD	37	32/37 (87)	1:17.5	33/37 (89)	45	41 (17-72)	19/40 (48)†
Controls	42	40/42 (95)	1:2.8	26/42 (62)	42	39 (15-70)	37/40 (93)‡
MS	28	26/28 (92)	1:2.1	26/28 (93)	28	38 (15-69)	2/27
OND	14	14/14 (100)	1:3.7	0/14 (0)	14	45 (20-70)	1/13

Diagnoses in the MS group included relapsing-remitting MS in 26; secondary progressive MS in 1; and primary progressive MS in 1. Diagnoses in the OND group included acute demyelinating encephalomyelitis in 4; non-longitudinally extensive transverse myelitis in 1; brain stem encephalitis of unknown aetiology in 1; autoimmune cerebellitis in 1; Herpes simplex virus encephalitis in 1; CNS lymphoma in 1; primary angiitis of the CNS in 1; Behçet's disease in 1; benign paroxysmal positional vertigo in 1; hydrocephalus aresorptivus in 1; and spinal disc prolaps in 1. NMO was diagnosed according to reference [39]. LETM was defined as myelitis extending over three or more segments as demonstrated by magnetic resonance imaging. MS was diagnosed according to reference [40]. † Treatments in the remaining cases included oral steroids, intravenous methylprednisolone, azathioprine, methotrexate, and cyclo-phosphamide; in 5 cases no exact data on the treatment status at time of LP were available. ‡ In 2 cases, no exact data on the treatment status was available. LETM = longitudinally extensive transverse myelitis; MS = multiple sclerosis; NMO = neuromyelitis optica; n.d. = not determined; NMOSD = neuromyelitis spectrum disorders; ON = optic neuritis; OND = other neurological diseases

## Results

### AQP4-Ab CSF status

AQP4-IgG was detected in 21/31 (68%) CSF samples from AQP4-Ab seropositive NMOSD patients. 0/14 CSF samples from AQP4-IgG seronegative NMOSD patients and 0/42 control samples were positive for AQP4-IgG. No significant difference regarding CSF AQP4-Ab frequency was found between acute relapses of ON (75%) and myelitis (59%). Detailed results are given in Table 2.

**Table 2:** Clinical findings, AQP4-Ab status in serum and CSF, and median AQP4-Ab serum titres in the various disease groups.

Diagnosis	No of CSF samples	Acute attack at time of LP (%)	AQP4-Ab, serum (%)	AQP4-Ab, CSF (%)	Median serum titre (range; N)
NMOSD	45	29/40 (73)	31/45 (69)	21/45 (47)	1000 (10-12500;26)
NMO	26	16/22 (73)	16/26 (62)	11/26 (42)	1000 (125-7000;12)
LETM	8	6/8 (75)	7/8 (88)	5/8 (63)	250 (62.5-12500;7)
ON	11	7/10 (70)	8/11 (73)	5/11 (45)	250 (10-7000;7)
*Relapse*	29	29/29 (100)	20/29 (69)	17/29 (59)	1000 (250-12500;20)
*Remission*	11	0/11 (0)	7/11 (64)	1/11 (9)	187.5 (10-250;6)
*AQP4-Ab seropositive*	31	20/31 (65)	31/31 (100)	21/31 (68)	1000 (10-12500;26)
*AQP4-Ab seronegative*	14	9/14 (64)	0/14 (0)	0/14 (0)	Negative
Controls	42	31/38 (82)	0/42 (0)	0/42 (0)	Negative
MS	28	22/28 (79)	0/28 (0)	0/28 (0)	Negative
OND	14	9/10 (90)^§^	0/14 (0)	0/14 (0)	Negative

AQP4-Ab = aquaporin-4 antibody; CSF = cerebrospinal fluid; LETM = longitudinally extensive transverse myelitis; MS = multiple sclerosis; NMOSD = NMO spectrum disorders; NMO = neuromyelitis optica; ON = optic neuritis; OND = other neurological diseases. ^§ ^Not applicable in four patients with non-inflammatory neurological diseases.

### AQP4-Ab serum titres

**Figure 1:**
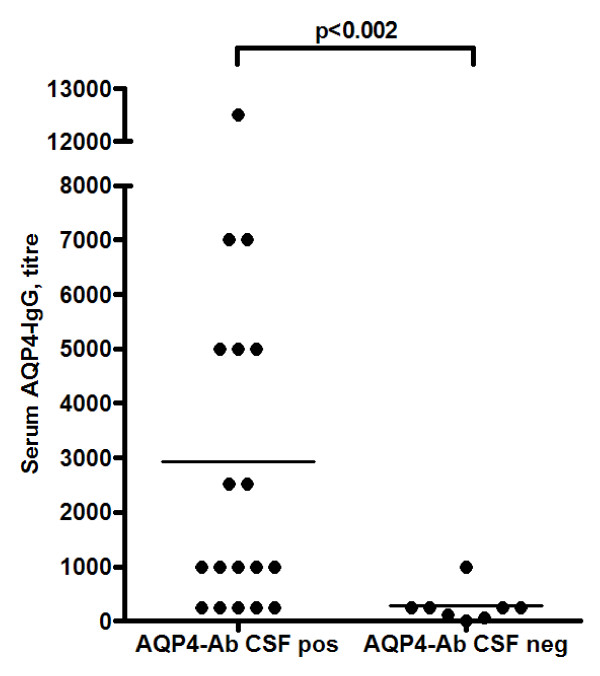
**Distribution of AQP4-IgG serum titres according to AQP4-Ab CSF status**. AQP4-Ab IgG serum titres were significantly higher in AQP4-Ab CSF-positive patients.

Serum AQP4-IgG titres were determined in 26/31 NMOSD samples and were higher (median 1:1000; range, 1:250-1:12.500) in CSF AQP4-IgG-positive patients (n = 18) than in CSF AQP4-IgG-negative patients (1:250; 1:10-1:1000; n = 8) (p < 0.002; Mann-Whitney test). See Figure 1 and Table 2 for details.

### Blood-CSF barrier function

Disturbances of the blood-CSF barrier as indicated by elevated Q_Alb _were present in 11/18 (61%) AQP4-Ab serum and CSF positive NMOSD samples but only in 1/8 (13%) of the AQP4-Ab seropositive but CSF-negative NMOSD samples analysed (p = 0.036; Fisher exact test). In the AQP4-Ab seronegative group, blood-CSF barrier dysfunction was present in 6/9 (67%) cases. In the remaining cases not enough CSF or serum was available for Q_Alb _determination. No significant correlation between AQP4-Ab titres and Q_Alb _was found.

### Total IgG in the CSF and serum

Evidence for intrathecal synthesis of total IgG as indicated by either CSF-restricted OCBs or elevated Q_IgG _was present in 32.5% (13/40) NMOSD, and was slightly more frequent in AQP4-Ab seronegative NMOSD samples (50%, or 6/12) compared to seropositive samples (25%, or 7/28). No significant correlation with the AQP4-Ab CSF status was found; while 100% (7/7) of the samples with intrathecally produced total IgG that were obtained from AQP4-Ab seropositive patients were positive for CSF AQP4-Ab, also 57.1% (12/21) of the CSF samples from AQP4-Ab seropositive patients with neither OCBs nor Q_IgG _elevation harboured AQP4-Ab (p = n.s.; Fisher exact test). Also, Q_IgG _and AQP4-Ab titres showed no significant correlation.

### AQP4-Ab AI

Intrathecal production (IP) of AQP4-IgG as defined by an AI > 4 was found in only 1/23 NMOSD samples analysed (4.3%) (AI_AQP4 _= 7). This patient had experienced a third attack of ON 12 days prior to LP (first LETM 10 months later) and had received high-dose methylprednisolone until nine days and low-dose steroids until five days prior to LP. The patient had co-existing acetylcholine receptor-antibody positive myasthenia gravis (treated with pyridostigmine) and thyroglobulin and thyroid peroxidase antibody positive thyroiditis. Clinical and laboratory findings at time of LP were otherwise unremarkable when compared to the remaining patients (Table 3). The sample was positive for OCBs, and Q_IgG _(5.9; Q_lim(IgG) _= 5.3; intrathecal IgG fraction, 8.7%) and Q_Alb _(7.2; age-adjusted upper reference limit = 5.5) were both elevated. A follow-up sample taken from the same patient one year later during another relapse of ON and still prior to initiation of long-term immunosuppression did not show evidence of AQP4-Ab IP anymore, though the antibody was still detectable in the CSF. In the remaining cases, not enough CSF and serum for Q_IgG _and Q_Alb _determination was available, so that AQP4-AI could not be assessed (n = 5), or CSF titres that would indicate a positive AQP4-AI were below 1:1 (n = 3), so that testing was not possible (all of the latter samples were negative at 1:1 dilution).

**Table 3:** Clinical and laboratory features according to AQP4-AI status.

	AQP4-Ab AI pos	AQP4-Ab AI neg
No of CSF samples	1	22
Sex	Female	All female
Age	22	40 (14-72)
Diagnosis at time of LP	rON*	NMO in 11; LETM in 6; ON in 5
IS/IM treatment at time of LP	Steroids until 5 d before LP	11/22
Disease duration in months, median	19.4	20.5 (0.1-120)
Acute attack at time of LP	Yes	19/22
AQP4-Ab serum titre, median (range)	1:1000	1000 (250-12500)
QIgG, median (range)	5.85	4.8 (1.5-16.1)
Intrathecal synthesis of total IgG	Yes	6/22
QAlb, median (range)	7.17	6.7 (3.2-31.1)
Blood-CSF barrier dysfunction	Yes	11/22

* Developed NMO 10 months later. AI = antibody index; IS/IM = immunosuppressive/immunomodulatory; NMO = neuromyelitis optica; LETM = longitudinally extensive transverse myelitis; LP = lumbar puncture; QAlb = albumin CSF/serum ratio; QIgG = IgG CSF/serum ratio; rON = recurrent optic neuritis.

### Impact of disease activity

17/20 (85%) CSF samples from AQP4-Ab seropositive NMOSD patients obtained within 30 days after onset of relapse were AQP4-Ab positive but only 1/7 (14.3%) taken during remission (p = 0.0017; Fisher exact test, 2-tailed). Similarly, 17/18 (94.4%) samples positive for CSF and serum AQP4-Ab were taken during acute relapse; by contrast, only 3/9 (33.3%) AQP4-Ab CSF-negative but AQP4-Ab seropositive samples were obtained during relapse (p = 0.002; Fisher exact test). The median serum AQP4-Ab titre of those NMSOD samples taken during relapse (1:1000; range, 1:250-1:12500; n = 20) was higher than in NMOSD samples obtained during remission (1:187.5; 1:10-1:250; n = 6) (p = 0.0008; Mann-Whitney test). AQP4-Ab serum titres did not differ markedly between acute relapses of myelitis (median, 1:1000; n = 13) and ON (median, 1:2500; n = 6), and were relatively high in the only patient with acute relapse of simultaneous optic neuritis and myelitis (1:5000). Serum AQP4-Ab titre in the single patient with evidence for AQP4-Ab IP was 1:1000. IP of total IgG was found in 7/20 (35%) AQP4-Ab seropositive samples obtained during relapse but in 0/7 taken during remission. Disruption of the blood-CSF barrier was found both with AQP4-IgG seropositive samples taken during relapse (10/20; 50%) and with some of those obtained during remission (2/6; 33%). No significant differences between acute relapses of myelitis and acute relapses of ON were found regarding the frequency of total IgG IP or of blood-CSF barrier disruption.

### Impact of disease duration

Median disease duration at time of LP was shorter (24.5 v 4.1 months) in the AQP4-Ab seronegative group (p = 0.056; Mann-Whitney test). Among seropositive patients, those positive for CSF AQP4-Ab had a longer disease duration (52.8 vs 19.45 months; p = 0.041). No correlation between serum titres and disease duration or time since relapse onset was found.

### Impact of treatment status

Median serum AQP4-Ab titres did not differ significantly between untreated (n = 12; 1:625; range, 1:62.5-1:12500) and treated (n = 14; 1:1000; 1:10-1:7000) NMOSD patients nor did the AQP4-IgG CSF positivity rate (64.3% and 75%, respectively). The only patient with positive AI_AQP4 _had received steroids until five days before LP.

### Longitudinal analysis

In total, 8 follow-up samples from 8 patients with NMOSD were examined for AQP4-Ab (median latency, 381 days). No patient who was initially positive for serum AQP4-Ab was negative at follow up; the only patient negative at first testing was also negative at follow-up. However, the disease status (relapse or remission) at first and second LP was identical in all cases. Also, in all cases, CSF was positive for AQP4-Ab in both samples, if samples were taken during relapse, or negative, if samples were taken during remission; the only exception was one AQP4-Ab seropositive sample that was negative for CSF AQP4-Ab during a relapse of ON but positive during a relapse of myelitis.

## Discussion

In this study we systematically evaluated the frequency of AQP4-IgG in the CSF and serum of Caucasian patients with NMOSD. Using a CBA employing recombinant human AQP4, we found CSF AQP4-IgG in ~70% of AQP4-IgG seropositive NMOSD samples, but in none of the MS or OND controls.

AQP4-IgG CSF positivity was associated with higher AQP4-IgG serum titres and with dysfunction of the blood-CSF barrier. Moreover, AQP4-IgG was more frequently detectable during relapse (p = 0.0017). The latter finding most likely reflects an increase in serum AQP4-IgG titres during relapse, since no evidence of intrathecally produced AQP4-IgG was found in almost all cases analysed. Serum titres were indeed significantly higher during relapse than in remission in our patients (median, 1:1000 vs 1:187.5) (p = 0.001), which is in line with previous studies [13,20]. Interestingly, the cut-off serum AQP4-IgG titre that predicted CSF AQP4-IgG positivity (1:250) was similar to that found in Japanese patients with opticospinal MS [13].

Serum titres >1:250 were associated with acute disease in all cases, and titres <1:250 with remission. In contrast, no clear correlation was found in case the serum titre was 1:250. It should be mentioned as a caveat, however, that the number of samples obtained during remission was relatively low in this study (as LP is mainly done for acute disease). In a previous study that included samples obtained over a period of up to five years, we could demonstrate marked variations over time regarding AQP4-Ab concentrations during remission with no general cut-off for relapse induction (though relapses were always preceded by an relative increase in AQP4-Ab levels). The latter finding might indicate differences in AQP4-Ab affinity and specificity between patients and over time, but inter- and intraindividual variations regarding T-cell activation, cytokine levels, or BBB function may also play a role.

Serum AQP4-Ab titres did not differ markedly between untreated and treated NMOSD patients nor did the AQP4-IgG CSF-positivity rate. This could be due to the fact that 11/14 samples from treated patients were taken during relapse, which was more commonly associated with high serum AQP4-Ab titres as well as with CSF AQP4-Ab.

Intrathecal AQP4-Ab production was present in only 1 out of 23 samples studied (4.3%). This sample was obtained during an acute relapse of ON. However, 20/23 AQP4-IgG CSF-positive samples with normal AI_AQP4 _values were also taken during relapse. AI_AQP4 _elevation seems thus not a suitable disease activity marker. The infrequency of AQP4-IgG IP suggests that in patients with NMOSD AQP4-Ab producing B cell clones usually reside in the systemic compartment. CSF AQP4-Ab might thus reflect passive diffusion of serum AQP4-Ab into the CSF. Accordingly, Takahashi et al. (2007), in a study on 12 Japanese patients, found that titres of CSF AQP4-IgG were almost proportional to serum AQP4-IgG in NMO, though, as a limitation, that study had not taken into account possible blood-CSF barrier disruption [13].

Although intrathecal AQP4-Ab production would then not be a prerequisite for inflammation in NMO, the single patient with a positive AI in our series (as well as a second recently published case with a slightly elevated AI[8]) indicates that intrathecal AQP4-Ab production can occur in NMOSD. However, the rarity with which it was detected argues against a major pathogenic function.

Unlike in MS, intrathecal total IgG synthesis in NMO does not persist over time [21-24]. It is of note that evidence for total IgG IP was present in 35% of seropositive samples obtained during relapse but in none of the seropositive samples obtained during remission. In line with this finding, Melamud et al., in a study so far only published as abstract, reported intrathecal total IgG synthesis in 45% of samples obtained in bout versus 0% in remission [24]. This might explain the low frequency of intrathecal total IgG production (17-33%) reported in the literature [1,21-23,25] compared to MS (95-100%). Similarly, AQP4-Ab might be present in the CSF only transiently as indicated by our finding of a higher AQP4-Ab CSF positivity rate during relapse. It is of note, however, that all eleven samples obtained at approximately (+/- 8 days) the same time from onset of relapse than the only AI positive sample (which was taken at day 12 after relapse onset), showed a negative AI, rendering it unlikely that timing issues are of high importance.

In our cohort, all CSF AQP4-IgG positive samples were also positive for serum AQP4-IgG, and AQP4-Ab serum titres were higher than AQP4-Ab CSF titres in all cases analysed. This would indicate that testing of CSF for AQP4-Ab in addition to serum AQP4-IgG testing is of limited benefit. In contrast, Klawiter et al. recently described three patients positive for AQP4-Ab in the CSF but not in the serum [12]. However, some constraints apply. First, a 1:128 serum dilution was used to test for AQP4-IgG instead of the standard 1:60 starting dilution [2,4,26,27]. To our experience from longitudinal studies in AQP4-IgG positive patients[20], serum samples may be positive even only at 1:10 dilution, depending on disease activity status or treatment. Secondly, the method used to test for AQP4-IgG, immunohistochemistry (IHC), has been demonstrated to be less sensitive compared to recombinant assays [9,13,14]. Thirdly, the authors discuss that interfering auto-antibodies could have led to false-negative results, a problem inherent to the IHC assay but not to the CBA. Therefore, we speculate that those samples might have been positive if a more sensitive and specific assay as well as lower serum dilutions had been applied.

The concept that pathogenic effects in NMOSD are predominantly brought about by blood derived AQP4-Ab is compatible with the unique localisation of the AQP4 antigen. AQP4 is mainly expressed in the astrocytic endfeet at the glial-endothelial interface [3]. Local BBB disturbances would thus be sufficient to render the antigen directly accessible to serum antibodies. In a recent animal study, induction of BBB damage by pretreatment with complete Freund's adjuvans was in fact sufficient to cause NMO-like lesions following passive transfer of AQP4-Ab positive sera [28].

However, severe BBB damage might not even be a prerequisite for lesion formation. The pial cell layer that separates the perivascular Virchow-Robin space of larger vessels from the glia limitans is fenestrated at the level of the arterioles and is widely missing at the level of capillaries, venules, and veins. In these areas, the AQP4-expressing astrocytic endfeet are directly exposed to antibodies leaving the microvasculature [29]. Importantly, the perivascular and subpial spaces harbour all cellular components required to mount local immune responses [30,31]. Moreover, serum AQP4-IgG could gain access to the CSF in the absence of BBB damage via regions of physiologically increased BBB permeability such as the circumventricular organs or by extracellular pathways, which provide a quasi-equilibrium between plasma and the fluids of the CNS [30,32,33]. In an animal model of NMO, injection of AQP4-Ab positive sera and human complement into the CSF indeed resulted in generation of NMO-like lesions in the absence of BBB disruption or pre-existing CNS inflammation [34]. In our study, seven patients had in fact normal Q_Alb _values but suffered from an acute attack, had high serum AQP4-Ab titres >= 1:250 (median, 1:1000), and were positive for CSF AQP4-Ab at time of LP. However, as a limitation, three of those patients were treated at time of LP, and in three the attack had started already 23, 28 and 30 days before LP. Moreover, Q_Alb _might not be sufficiently sensitive to reflect locally restricted BBB disruption, in particular in ON, which was present in 4 of the 7 patients at time of LP.

Finally, AQP4-Ab itself could induce BBB damage. Prolonged exposure to AQP4-IgG in the fenestrated perivascular and subpial spaces could lead to BBB disruption by gradual local inflammation or AQP4 internalisation, followed by exacerbation of the autoimmune response [30]. A number of recent *in vitro *and *in vivo *studies revealed that IgG from patients with NMO initiates endocytosis of AQP4, a process which alters the polarized expression pattern of AQP4 on the plasma membrane and, as a functional consequence, increases BBB permeability *in vitro *[35-37]. In AQP4 knock-out mice, the lack of AQP4 is mirrored by tight junction opening in brain microvessels, swelling of perivascular astrocytic processes, and BBB hyperpermeability [38]. Interestingly, disease activity was linked to higher CSF AQP4-Ab and to higher serum AQP4-Ab levels in this study and another,[13] indicating that CSF and/or serum AQP4-IgG exceeding a threshold value might be required to induce clinically relevant inflammation.

## Conclusion

In summary, our study demonstrates that (1) AQP4-IgG is detectable in the CSF in most AQP4-IgG seropositive NMOSD patients but not in that of patients with MS or OND; (2) that the presence of CSF AQP4-IgG in patients with NMOSD is positively associated with acute disease relapse within 30 days prior of LP; AQP4-IgG serum titres >1:250; and with blood-CSF barrier disruption; but not with treatment status or the type of acute clinical disease (myelitis or ON) at time of LP; (3) a lack of quantitative evidence for intrathecal synthesis of AQP4-IgG in most NMOSD patients. Our findings argue against the need to test CSF for AQP4-Ab if the corresponding serum is negative for the antibody or AQP4-Ab serum titres are below 1:250. Moreover, our results suggest that intrathecal production of AQP4-Ab may not be a prerequisite of disease activity in NMO.

## Competing interests

SJ, DF, FP, KR, RB, PR, RR, WK, and BW declare no competing interests. The cells used in this study were kindly provided by Euroimmun, Luebeck, Germany. KPW and CP are employees of Euroimmun. Euroimmun had no role in study design, data collection or analysis, preparation of the manuscript, or decision to publish.

## Authors' contributions

SJ conceived and designed the study. SJ, CP, and KPW were involved in carrying out the immunoassays. SJ, DF, FP, KR, RB, PR, RR, WK, KPW, and BW participated in CSF and data collection. SJ performed the statistical analysis and wrote the initial draft. SJ, DF, FP, KR, RB, PR, RR, WK, KPW, and BW participated in the preparation of the manuscript. All authors read and approved the final version of the manuscript.
